# Current Gaps in Survey Design and Analysis for Molecular Xenomonitoring of Vector‐Borne Neglected Tropical Diseases: A Systematic Review

**DOI:** 10.1111/tmi.70017

**Published:** 2025-08-05

**Authors:** Angus McLure, Tilahun Alamnia, Zhiwei Xu, Colleen L. Lau, Helen J. Mayfield

**Affiliations:** ^1^ National Centre for Epidemiology and Population Health The Australian National University Canberra Australia; ^2^ College of Medicine and Health Sciences Bahir Dar University Bahir Dar Ethiopia; ^3^ School of Medicine and Dentistry Griffith University Gold Coast Australia; ^4^ Centre for Clinical Research, Faculty of Health, Medicine and Behavioural Sciences The University of Queensland Brisbane Australia

**Keywords:** lymphatic filariasis, molecular xenomonitoring, neglected tropical disease, onchocerciasis, vector surveillance

## Abstract

**Objectives:**

Molecular xenomonitoring is a surveillance method for vector‐borne diseases where vectors are tested for molecular pathogen markers. Testing is typically on pools (groups) of vectors. Molecular xenomonitoring is a sensitive and efficient complement to human‐based surveillance. However, existing statistical guidance for the appropriate design and analysis of molecular xenomonitoring surveys has key gaps. We reviewed the literature to understand the common objectives, survey designs, and analysis methods for molecular xenomonitoring surveys for two vector‐borne neglected tropical diseases: lymphatic filariasis and onchocerciasis.

**Methods:**

We searched peer‐reviewed literature for studies published between 1999 and 2022 that presented the results of surveys that collected vectors in field surveys and used a molecular test for the presence of the causative pathogens for lymphatic filariasis and onchocerciasis.

**Results:**

Out of 1225 works identified in the database search, a total of 76 studies (lymphatic filariasis: 45; onchocerciasis: 31) across 30 countries were included in the review. The five most common objectives were determination of elimination status after mass drug administration, comparison of vector and human infection indicators, evaluation of an intervention, comparison of vector collection methods and comparison of laboratory techniques. Nearly all studies used a cluster or hierarchical sampling framework to collect vectors (72/76), but very few studies accounted for this in their designs (2/76) or analysis (1/76). While few studies justified the number of vectors included in each pool (5/76), nearly all studies accounted for pooled testing when calculating pathogen prevalence from results (69/76). Few studies justified the number or selection of collection sites or total sample size (16/76).

**Conclusions:**

Published molecular xenomonitoring surveys for lymphatic filariasis and onchocerciasis had varied objectives, study designs and analysis methods, but proper consideration of survey design was frequently missing from the analysis. There is a need for statistical tools and guidance to enable appropriate design and analysis of molecular xenomonitoring surveys while accounting for disease, objective and context‐specific considerations.

## 1 Introduction

Neglected tropical diseases (NTDs) remain a major cause of mortality and morbidity, disproportionality affecting low‐income countries [1]. Several of these diseases are the focus of targeted global elimination campaigns that rely on mass drug administration (MDA) as the primary intervention [2]. For these programmes to meet their objectives, efficient and effective surveillance tools that can detect evidence of infection or transmission in specific populations and locations are crucial to facilitate informed, evidence‐based decision making. This requirement is particularly critical as these programmes approach their end game, when the prevalence of the diseases becomes very low and often more focal [3, 4, 5, 6, 7]. Molecular xenomonitoring (MX), the collection of disease vectors and other biting invertebrates and testing them for the presence of molecular markers of the pathogen, is one of a range of strategies that can provide evidence of pathogen presence and transmission potential in an area [8].

MX studies can support several specific objectives of disease surveillance, including demonstration of pathogen presence/absence in a study area [9, 10, 11, 12, 13, 14, 15], comparing the prevalence of the pathogen marker in the vectors to a threshold to inform programmatic decisions [5, 16, 17, 18], or comparing the prevalence of the pathogen marker in two samples of vectors, for example, before and after an intervention [19, 20, 21, 22, 23]. When applying MX to a new pathogen, vector, or setting, initial studies will focus on validating the tool and identifying effective ways of capturing and testing the vectors [24, 25, 26, 27]. A key part of this validation is establishing the most appropriate survey designs and how best to analyse results [28, 29].

MX is often used as a complement to disease surveillance in humans [9, 10, 12, 13, 21] and has its own set of advantages and disadvantages. MX can be less intrusive than human surveillance methods, which often involve taking blood or tissue samples. Though highly dependent on local conditions, including weather and climate, it is often possible to sample very large numbers of vectors, far more than the number of humans who could be tested for a similar amount of survey effort [21, 25]. To reduce time and costs, vectors are often tested in pools (groups) rather than individually, with a single positive or negative test result for the whole pool. Though not an issue for surveys attempting to establish the presence or absence of a pathogen in a vector population, pooled testing leads to a loss of information when it comes to estimating the prevalence of the pathogen [30].

There are several characteristics of MX surveys that complicate their design and analysis. In addition to the pooled testing methodology, MX surveys often utilise cluster designs or a hierarchical sampling framework, with the vectors collected at a number of collection sites across the area of interest. Both cluster sampling and pool testing can reduce the total cost or effort of the survey but reduce the effective sample size and complicate design and analysis [31]. Furthermore, different vector collection methods may catch biting insects of multiple species and different blood‐fed statuses, with different degrees of vector competency, feeding preferences, or exposure to infective vertebrate hosts, complicating the interpretation of pathogen detection or absence.

Efficient and appropriate survey designs and analysis plans are therefore important to maximise the information gained for minimal cost. Early consideration of design and analysis is critical to provide the best evidence to support decision making in disease elimination programmes. However, there is relatively little published research that assesses the appropriateness of common MX survey designs. While there are WHO guidelines for the use of MX for some NTDs, these guidelines do not cover all considerations [32, 33, 34]. Understanding the common objectives, design and analysis of MX surveys is the key first step before this gap can be addressed.

In this systematic review, we focus on the use of MX for two vector‐borne NTDs: onchocerciasis, transmitted by biting blackflies of the *Simulium damnosum* complex; and lymphatic filariasis (LF), transmitted by mosquitos of the genera *Aedes, Culex, Anopheles* and *Mansonia*. Both diseases are caused by filarial parasites where the adult stages infect human hosts for many years and produce offspring that must pass through a vector host before maturing. Both diseases cause major disability in a minority of human cases and are targeted for global elimination using MDA. MX has been used extensively for both diseases, and the WHO endorses the use of MX across both elimination programmes; however, while evidence from MX surveys is required by WHO to certify the elimination of onchocerciasis, the same is not true for LF programmes [32, 33, 35]. There are several existing reviews of MX for these two NTDs. Pilotte et al. provide an excellent overview of the methods, strengths and operational research gaps for MX [8]. Pryce and Reimer and Pryce et al. evaluated the sensitivity of MX surveys to detect locations of people with microfilaremia [36, 37]. Reimer and Pryce examined the effect of vector sampling methods and vector genus on the prevalence of mosquitos positive for filarial DNA [38]. Unlike previous reviews, we take a statistical perspective on MX survey design and analysis. We ask the following questions of MX surveys published in peer‐reviewed literature:

- What were the *objectives* of MX surveys?
- What survey *designs* (e.g., site selection, sample sizes, pooling strategies) were used for MX and how are these designs selected?
- How were data from MX surveys *analysed*?
- Were the above considerations (objective, design and analysis) well aligned and how could this alignment be improved?

## 2 Methods

An initial literature search was performed on PubMed database for articles published between January 1999 and September 2022 (date of search). The criteria for inclusion were titles or abstracts that included a term related to MX (molecular xenomonitoring OR mosquito surveillance OR vector surveillance) as well as a term related to one of the target diseases (lymphatic filariasis OR onchocerciasis). Titles were imported into Covidence for screening [39]. Title and abstract screening were conducted by two reviewers (T.A. and Z.X.) with conflicts reviewed by discussion and consensus. Full text screening was conducted by two reviewers (T.A. and A.M.).

The following types of studies were excluded: reviews without new analysis/presentation of data; studies that did not collect vectors or other biting insects (e.g., if only humans were sampled); studies where no insects were tested using a molecular test for the presence of the pathogen; and studies that did not present data from a field survey (e.g., simulation studies; studies to validate molecular testing on experimentally infected vectors); articles not in English. The following types of studies were *not* excluded unless they also met one of the above exclusion criteria: reanalysis/secondary analysis of data; studies that tested insects but found all to be negative for the pathogen marker; studies where some vectors were tested individually (rather than in pools).

Data extraction was conducted by two reviewers (H.J.M. and A.M.) and included the following variables: year of publication; disease (LF or onchocerciasis); country(ies) or territory(ies) where insects were collected; study objective(s); whether the study used a hierarchical survey design and details including site selection; total number of insects caught and/or tested; justification of the sample size; number of pools tested; whether any pools tested positive for pathogen DNA; pooling strategy and justification for this strategy; whether insect species were separated before pooling and testing; software used to analyse survey data; whether analysis of data was hierarchical and details including the levels at which the analysis was conducted.

To help classify studies with complex or multiple objectives, each study was classified as addressing one or more of the following: validation of elimination following an MDA program; evaluation of an intervention with pre and post surveys; comparison of MX indicators to human‐based indicators; comparison of laboratory techniques for detecting pathogen DNA in mosquitos; and comparison of vector collection methods. Where appropriate, extracted results were summarised using counts and percentages in R [40].

## 3 Results

### 3.1 Included Studies by Country and Disease

**FIGURE 1:**
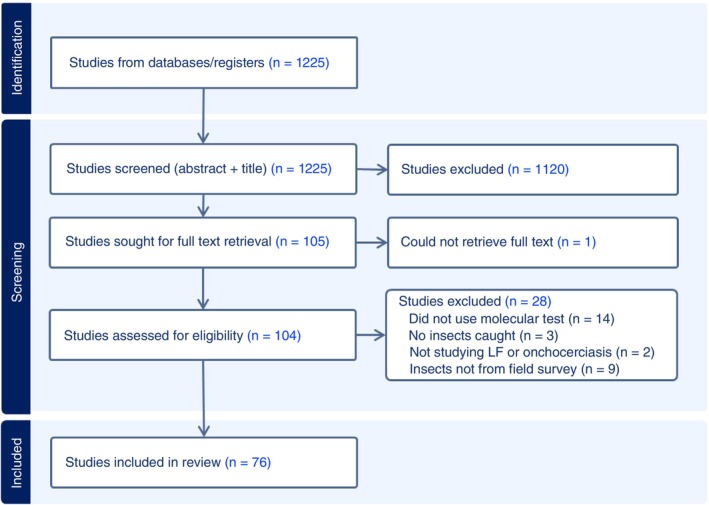
Summary of number of papers identified and screened, including reasons for exclusion.

The database search identified 1225 unique studies. Of these, 1120 studies were excluded based on abstract and title. Of the 105 remaining studies, one was excluded as we could not retrieve the full text, two studies were excluded because they were not on LF or onchocerciasis, three studies were excluded because no insects were caught, 14 were excluded because they did not use a molecular test to detect pathogen DNA in insects, and nine were excluded because they did not include insects from field surveys (Figure 1). A total of 76 studies were included in the review: 31 on onchocerciasis [5, 12, 13, 16, 18, 23, 41, 42, 43, 44, 45, 46, 47, 48, 49, 50, 51, 52, 53, 54, 55, 56, 57, 58, 59, 60, 61, 62, 63, 64, 65] and 45 on LF [7, 9, 10, 11, 14, 15, 17, 19, 20, 21, 22, 24, 25, 26, 27, 28, 29, 66, 67, 68, 69, 70, 71, 72, 73, 74, 75, 76, 77, 78, 79, 80, 81, 82, 83, 84, 85, 86, 87, 88, 89, 90, 91, 92, 93]. No study covered both LF and onchocerciasis. A table with all included studies along with their key characteristics is included in Supporting Information: File A.

The included studies were conducted across 30 countries, with LF studies in 20 countries and onchocerciasis studies in 15 countries. Five studies were undertaken across more than one country, and five countries had studies for both diseases. Sri Lanka had the most studies for LF (seven) and Mexico had the most studies for onchocerciasis (seven). Nigeria had five studies: one for LF and four for onchocerciasis. By region, the largest number of studies was conducted in Africa (20 LF and 15 onchocerciasis). The remaining 16 studies of onchocerciasis were all in the Americas, while the remaining studies of LF were distributed across Asia (14) and the Pacific (8), with three studies in the Americas (Brazil).

### 3.2 Study Objectives

**FIGURE 2:**
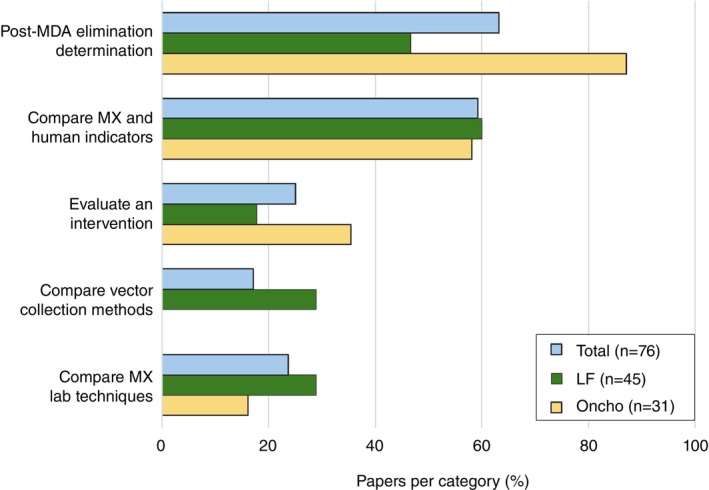
Frequency of the five most common objectives of MX surveys in the included studies by disease and overall. Some studies had multiple objectives. Values are provided in Table S1. LF: lymphatic filariasis; Oncho: onchocerciasis.

Studies had a wide range of objectives. The most common objective overall (48/76 studies) and for onchocerciasis studies (27/31 studies) was determination of elimination status (i.e., validation) post‐MDA. Most studies (LF: 27/45; onchocerciasis: 18/31; overall: 45/76) also made comparisons between indicators of infection markers in humans (e.g., detection of microfilaria or antigens) and MX indicators. One quarter of studies (19/76) evaluated interventions by comparing MX indicators in surveys before and after the intervention(s). Thirteen studies, all for LF, compared different insect collection techniques. Eighteen studies, predominantly on LF (13), compared different lab techniques for detecting pathogen DNA in the insects. Though objectives were broadly similar across the two diseases, onchocerciasis studies were more often focused on programmatic use of MX (evaluating elimination and post‐MDA elimination determination), while LF studies were more often focused on establishing methods (comparing vector collection methods and lab techniques). The number of studies addressing each of the five most common objectives is listed in Figure 2.

### 3.3 Post‐MDA Elimination Determination

Nearly all studies of onchocerciasis (27/31) and half of LF studies (21/45) aimed to evaluate progress towards elimination in settings where multiple rounds of MDA had been conducted. Many of these onchocerciasis studies compared the proportion of infective and infected blackflies to thresholds set by the WHO (0.1% in parous flies or 0.05% in all flies) [32] to validate elimination status and evaluate the need for further MDA. Most onchocerciasis studies with this objective confirmed that prevalence had reached very low levels, with about half (14/27) reporting no detections of positive blackflies, and a number of studies with longitudinal sampling reporting no detections in the latter years [16, 60]. For LF, there are only provisional WHO targets for thresholds to inform decisions to stop or start MDA using MX markers, with the value dependent on the dominant vector species in the population: *Culex* 0.25%; *Anopheles* 1%; and *Aedes* 0.1% [33]. Two studies in this review proposed thresholds for populations with *Culex‐dominated* transmission: 0.25% [22] or 1.0% [85]. These values have then been used in a number of subsequent studies that compared prevalence to one of these thresholds [7, 25, 28, 77, 81]. Other studies in this review [20, 21, 22, 72, 77, 81, 84] cited alternative thresholds for *Culex* (0.5% [94]) and *Anopheles* (0.65% [95]) dominated populations. Some LF studies evaluating progress towards elimination in post‐MDA settings reported no detection of filarial DNA in mosquitoes (6/21 studies) [9, 11, 24, 67, 72, 79].

### 3.4 Comparing MX and Human Indicators

MX studies were often conducted alongside or compared to results of surveillance of human indicators in the same geographical area: 18/31 (58%) onchocerciasis studies and 27/45 (60%) of LF studies. In these studies, human participants would be screened for indicators of current or past infection, including filarial antigens, anti‐filarial antibodies and detection of microfilaria in skin or blood using microscopy. For many of these studies, particularly onchocerciasis studies in which the primary objective was to evaluate progress towards elimination, the primary comparison was between the indicators (human and entomological) and respective thresholds, with the two types of indicators providing independent lines of evidence for or against elimination. However, in a number of LF studies, human and entomological indicators were compared in terms of sensitivity to detect low prevalence of pathogen markers [21, 25, 29, 77, 85, 86]. Some studies also compared the cost of human surveillance and MX, such as Subramanian et al. [25] that found that MX had a similar cost but was more sensitive for detecting markers of LF infection/transmission in a population than standard transmission assessment surveys (TAS) in children.

### 3.5 Evaluating an Intervention

Of the studies that evaluated an intervention (19/76), all but one evaluated MDA, with a single study (LF) evaluating the effect of bed‐nets [19]. In some studies, the intervention (MDA) was conducted repeatedly with surveys before, between and after rounds. In these studies, the prevalence of filarial markers in insects or other entomological markers was compared between repeated surveys. Most studies evaluating an intervention (LF: 7/8; Onchocerciasis: 7/11) used MX alongside human indicators. Studies overwhelmingly found that when there was any trend between pre‐ and post‐surveys, human and MX indicators showed the same trend. Notably, McPherson et al. [21] reported that within a year of the intervention (MDA), the prevalence of filarial DNA had significantly declined, but did not detect a significant decline in filarial antigen prevalence in humans.

### 3.6 Comparing Vector Collection Methods or Lab Techniques

All studies comparing vector collection methods came from the LF literature and mostly focused on the yields of mosquitos caught by different types of traps or human landing catches. One study [29] considered the number of collection sites, comparing the estimates of filarial DNA prevalence when conducting intensive sampling at a few sites versus collection of the same number of mosquitos at a larger number of sites. Studies comparing laboratory techniques were also mostly of LF (13/18) and usually compared a molecular test such as PCR detection of filarial DNA to dissection of insects for microscopy. However, a number of studies compared molecular techniques to each other, for example, comparing real‐time PCR, LAMP and dissection [23, 82]; comparing simple and multiplex PCR [69]; or comparing novel high‐throughput automated PCR systems to existing PCR methods [61]. The primary comparison was that of sensitivity to detect the pathogen, with studies concluding that molecular techniques were as sensitive [23, 56] or more sensitive [66, 68, 92] than microscopy. Two studies also compared the cost of detection methods, either dissection versus PCR [64], or dissection versus PCR versus PCR‐ELISA [92].

### 3.7 Other Objectives

Other less common objectives not included in Figure 2 were also found. Several studies used MX to identify or exclude the possibility of transmission in areas not previously known to be endemic and had no history of MDA [14, 41, 75, 76, 81]. Some studies, primarily of onchocerciasis, reported longitudinal post‐MDA surveillance attempting to detect signs of recrudescence or establish that prevalence continued to decline after cessation of MDA [9, 42, 51, 57, 59, 60, 62, 63, 85, 86]. Many studies measured entomological indices beyond the detection of pathogen DNA. Annual biting rates were sometimes calculated in LF studies [82] and often calculated for onchocerciasis studies [12, 16, 46, 50, 54, 60, 62] as an intermediary to calculating the number of infective bites per person per year. Other studies focused on detailed analyses of the composition of vector species present [80], compared vectorial capacity between present species [44], measured the prevalence of insecticide resistance [80], or vector dispersion using mark‐release‐recapture experiments [24]. Some studies tried to understand the environmental factors influencing vector abundance as measured through counts (number of insects trapped) [78], capture rates (vectors trapped per trap per unit time) [83], biting rates (bites per person per unit time in human landing catches) [12] and other indices. Some considered factors influencing vector abundance in a region [23] or around households [78], while others examined seasonal variations in vector abundance and prevalence of filarial DNA [12, 23, 83]. One study attempted to identify correlation between environmental factors (rainfall), vector abundance and prevalence of filarial DNA in vectors [88]. A few studies reported the total and itemised costs for MX surveys [11, 25].

## 4 Survey Designs

### 4.1 Sampling Framework

The included studies employed a wide variety of survey designs to collect, pool and test disease vectors. Sample designs for the collection of insects were often complex and studies used different terms to describe the key elements. We briefly set out some generic terms to facilitate the discussion of these designs. Vector populations are dynamic with short lifespans and population sizes that fluctuate with weather conditions; therefore, the units of selection in survey designs cannot be individual insects, but the individual locations at which insects are collected, which we call *collection sites*. When a survey of a population involves more than one collection site, we call this *hierarchical sampling*. All hierarchical MX sampling designs involve at least two *final stages*: (a) selection of collection sites and (b) collection of insects. However, designs can involve additional *earlier stages*. In a 3‐stage design, there is one earlier stage in which the study area is divided into *stage‐1 areas* (e.g., villages) some of which are selected; in the two final stages (a) collection sites are selected from each selected stage‐1 area and (b) insects are collected from each collection site. In a 4‐stage design, the selected stage‐1 areas are further divided into smaller stage‐2 areas (e.g., neighbourhoods within villages) and some of the stage‐2 areas are selected for the two final stages.

**FIGURE 3:**
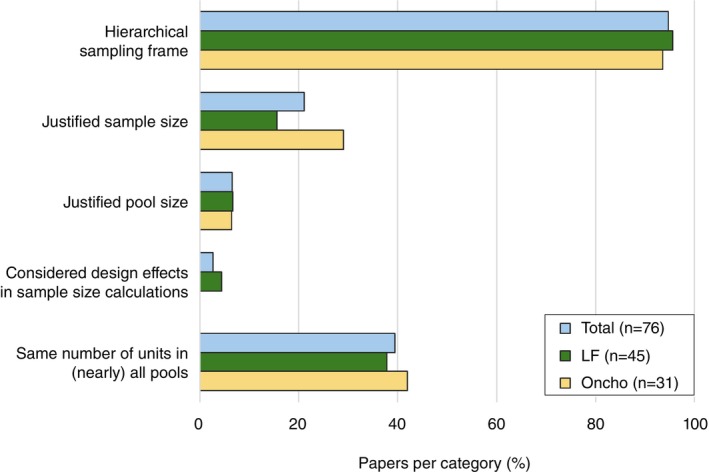
Frequency of key design choices and justifications for the MX surveys in the included studies by disease and overall. Values are provided in Table S2. LF: lymphatic filariasis; Oncho: onchocerciasis.

Nearly all the surveys (onchocerciasis: 29/31; LF: 43/45) utilised a hierarchical sampling framework, with the sampling framework being unclear for three studies [64, 67, 69], and one onchocerciasis study collecting insects from a single collection site [23] (Figure 3). In one study, vectors were collected from a single collection site in each stage‐1 area [41]. However, the majority of studies had multiple collection sites for each area selected in earlier stages.

### 4.2 Selection of Collection Sites and Sampling Areas

The selection of collection sites and stage‐1 or stage‐2 sampling areas was not clearly and completely described in many studies. In some studies, the areas in earlier stages of sampling were selected non‐randomly (e.g., villages, rivers, cities), with more than one collection site (traps or human landing catches) in each study area of interest [10, 12, 14, 22, 42, 68, 82, 83, 90, 93]. Other studies randomly or systematically selected earlier stage areas (e.g., village or a small public health unit) [28, 29, 70, 85, 89]. Site selection was randomised or systematic in some studies [7, 17, 21, 78], and purposive in others, usually targeting high‐risk locations where infected humans or vectors had been identified previously [53, 55] but sometimes based on accessibility [54]. Some surveys used a mix of purposive and randomised selection at the different sampling stages. For example, Derua et al. purposively selected villages and hamlets within villages to maximise ease of access and mosquito collections, but three households were randomly selected from each hamlet for vector collection [68].

More than half of the surveys conducted MX surveillance alongside surveillance in humans (onchocerciasis: 18/31; LF: 27/45) (Figure 3). For onchocerciasis studies, vector collection sites were often located near breeding sites (riverbanks) rather than households [23, 41]. However, many MX surveys, especially LF surveys, were conducted in or near homes, including concurrent MX and human surveillance studies [9, 21, 84] and MX‐only studies [28]. In a few studies, collection sites were chosen to be very close to the households of participants in the human surveys [21, 84]. In others, extensive sampling of households in both the human and entomological studies meant some household locations were included in both arms of the study [22, 75]. In most studies, however, the human and MX sampling were co‐located at the earlier stages of sampling but not at collection sites, for example, the same villages or same public health units, but not the same households.

### 4.3 Sample Size of Insects

The number of insects collected was generally very large, but with substantial variability between included studies. In some studies, particularly LF studies, only a portion of all collected vectors was tested for filarial DNA using molecular methods, and in some cases this portion was less than 30% of the total [68, 76] or as little as 2% [80]. The number of insects examined with a molecular test was the most reliably reported and readily comparable measure of sample size between studies, being reported in all but one study [74]. Using this metric, the sample size of insects was generally smaller for LF studies (median: 7900; IQR: 3100–15,000) than for onchocerciasis studies (median: 31,000, IQR: 12,000–86,000). Onchocerciasis studies where one of the objectives was post‐MDA elimination determination generally had larger sample sizes than studies without this objective (median: 34,000, IQR: 15,000–97,000 vs. median: 11,000, range: 7500–13,000). LF studies that aimed to determine elimination status after MDA had similar sample sizes (median: 8500; range: 4000‐23,000) to LF studies without this objective (median: 5800; IQR: 2500‐15,000).

### 4.4 Pooling Schemes

Pooling schemes varied substantially between studies. Studies stratified insect pools by one or more variables (e.g., collection site, collection time, collection method, vector species), though these were not always clearly specified. Stratification methods included pooling by collection site and time, with the time interval sometimes as short as 1 h [16, 41, 66] but often longer [20, 25, 28, 69, 84, 88, 90]; or pooling by earlier stages of sampling (e.g., village or community) and method of collection, but combining samples from different collection sites [11, 12, 42, 55, 58, 81, 92]; or with different pooling strategies in different study years [21, 44]. Some studies chose not to separate insects by species for some or all of their surveys [44] or separated only to genus level [17, 76, 77, 81, 82]. In many onchocerciasis studies, heads and bodies of flies were separated. In some studies, only pools of heads were tested [5, 18, 48], or bodies were tested first, followed by testing heads only from collection sites with positive body pools [16, 57, 58, 59]. Though much less common, at least one LF study [73] divided mosquitoes into body segments to test heads separately from the thorax and abdomen.

The maximum number of insects per pool varied substantially between studies and was typically larger in onchocerciasis studies (median: 50, range: 20–300) than LF studies (median: 20, range: 1–30). In some studies, the number of units per pool was the same across all (or nearly all) pools, while in others a range of pool sizes was used. Of the onchocerciasis studies, 13 used a fixed pool size, 14 a variable pool size, and four studies were unclear. Of the LF studies, 17 used a fixed pool size, 23 a variable pool size, and five studies were unclear. Similarly, some studies capped the number and size of pools per collection site [72], not testing any collected insects beyond these limits.

### 4.5 Justification of Survey Designs

Few studies provided any justification for the survey design (e.g., sampling frame, site selection, sample size, pooling scheme), with others providing justifications for only some of the design choices. No studies provided justification for the number of insects per pool on statistical grounds. One LF study [79] cited a WHO handbook [34] which states that ‘a pool of 25 mosquitoes is often used for PCR processing in determining infection,’ and did not provide any other justification. Another LF study [82] stated that the choice of pool size was based on a previous study [96] that compared different pool sizes (range: 25–200), but used smaller pools in their own study (range: 5–20) without further justification. Two onchocerciasis studies validated the sensitivity and specificity of the molecular test using known positive and negative pools with a range of sizes before applying the largest verified pool size to the field survey component of their study [56, 61], however did not provide statistical justification for using the largest validated pool size.

Most studies (LF: 38/45; onchocerciasis: 22/31) provided no justification of sample sizes beyond trying to catch as many insects or vectors as possible (Figure 3). The most common justification for sample size in onchocerciasis studies (six studies) was to collect enough blackflies such that if all were negative, the one‐sided 95% confidence interval for prevalence would be less than 0.05% [12, 13, 42, 45, 62, 63], citing guidelines published by the WHO; however, these studies reported different numbers of flies (3900 or 6000) needed to achieve the same goal. Six LF studies [11, 20, 21, 72, 81, 82] justified their choice of sample size in terms of power to determine whether prevalence was below a given threshold value, and another LF study [28] set target sample sizes based on desired precision of the prevalence estimate. Two LF studies [21, 72] included design effects in their sample size calculations to account for hierarchical sampling designs, and a third study [28] specifically stated that they did not include a design effect (i.e., design effect of 1); however, none of these studies justified their choice of design effects. No onchocerciasis studies discussed the inclusion of design effects.

While some studies indicated the number of vector collection sites and set targets for the number of vectors to collect at each site to meet a desired sample size [20, 25], studies rarely discussed or justified the number of collection sites or the number of vectors per collection site on statistical grounds. A notable exception (an LF study by Rao et al.) compared different study designs with the same total sample size but a different number of collection sites and mosquitos per collection site, finding that point estimates of prevalence were similar across designs [29]. However, as this study did not account for clustering of infection at collection sites, and the effect of clustering on confidence intervals is greater when the number of units per collection site is larger [97], this may not have been a fair comparison of different sampling approaches.

## 5 Data Analysis

Nearly all studies used an analysis method that could estimate insect‐level prevalence from the pooled data (onchocerciasis: 30/31; LF: 39/45). In studies where all pools were negative (onchocerciasis: 15/31; LF: 9/45), analysis did not require specialised software to adjust for pooled testing. Most studies used the Poolscreen software [98] to make the appropriate adjustment for the pooled testing protocol (onchocerciasis: 30/31; LF: 28/45). Two studies used R packages: Takagi et al. [88] used binGroup [99] and McPherson et al. [21] used PoolTestR [100]. Two studies [50, 69] had a fixed pool size and a further two studies [19, 70] appear to have tested insects individually, in which case prevalence could be estimated with a simple formula and did not require specialised software. Three studies with positive insect pools [14, 27, 91] only reported pool‐level results, or did not clearly state whether the results were adjusted for pooling. While most studies reported confidence intervals for estimates of prevalence, some studies did not [23, 24, 50, 68, 69, 70, 71, 91, 92], especially where there were no positive insects detected in the study [15, 24, 67, 75, 79, 80] or subpopulation [73, 89]. While nearly all studies used a hierarchical sampling design and most studies estimated prevalence in an area by aggregating the test results from pools from multiple sites, only one study [21] adjusted prevalence estimates for clustering at collection sites, using the PoolTestR R package [101].

Many studies compared the prevalence of pathogen markers in insects from two or more samples. The samples could be from different areas [21, 28, 44, 47, 78], different timepoints in the same areas [7, 9, 12, 16, 23, 42, 52, 60, 62, 86, 90], different insect species [21, 44], different trapping methods [78], or different detection methods [66, 89, 92]. Some studies used common statistical tests to examine the difference in the proportion of positive pools between samples, such as the chi‐squared [28], Fisher's exact [56, 66], Kruskal‐Wallis [23] and *t*‐tests [78]. In some of these studies, there were different numbers of insects in each pool, sometimes with systematic differences between samples [7, 28, 66, 86], and as none of these tests account for pool size, differences in pool sizes between samples may have masked or exaggerated any true difference in insect‐level prevalence between samples.

**FIGURE 4:**
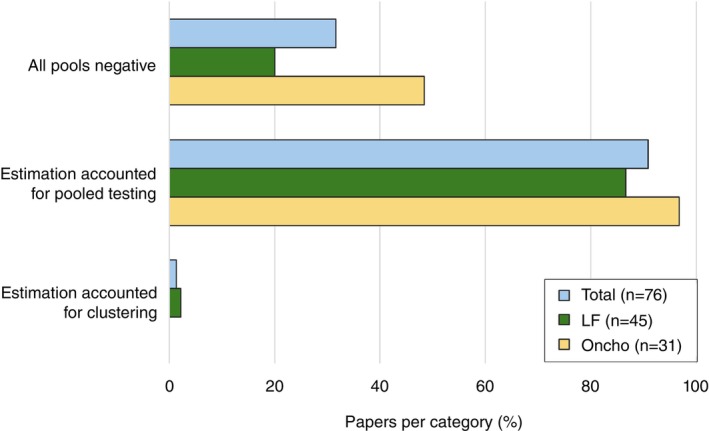
Frequency of key analysis choices and outcomes for the MX surveys in the included studies by disease and overall. Values are provided in Table S3. LF: lymphatic filariasis; Oncho: onchocerciasis.

In some studies, the confidence intervals around estimates were non‐overlapping and the samples could be considered independent, and therefore no further test was necessary to establish a difference between samples [47, 89]. However, even in some studies where a primary objective was to determine prevalence difference between two samples (e.g., before and after an intervention or different detection methods), there was often no quantification of the difference between the samples (e.g., prevalence ratios, prevalence differences), or no confidence interval for this difference, or no statistical tests for the significance of the difference [16, 52, 60, 89, 90]. Only one study [21] adjusted for the clustering or reported estimates of differences between samples (odds ratio for insect‐level positivity) together with intervals, using PoolTestR [100] for these calculations. A further study [44] determined a *p* value for the prevalence difference by finding the largest confidence level for which the pairs of confidence intervals did not overlap, adjusting for pool testing but not clustering of infection and collection sites (Figure 4).

## 6 Discussion

Our systematic review found that MX surveys for LF and onchocerciasis have diverse objectives, designs and analytic methods with many commonalities across the two diseases. Many of the differences in the literature between diseases exist because programmatic use of MX is more established and elimination programmes have further progressed for onchocerciasis compared to LF: onchocerciasis studies were more often focused on post‐MDA and post‐validation settings, using well established methods from WHO guidelines and large sample sizes to attempt to detect pathogens that were very rare or absent in the population, while LF studies were more often focused on settings where MDA is ongoing and establishing the proper sampling and testing protocols suited to the specifics of the pathogen/vector system suitable for each country. While it is beyond the scope of this review to make a detailed critique of the alignment between objectives, designs and analysis for each of the 76 included studies, we highlight common misalignments between objectives, survey design and analysis that we have identified and suggest tools or resources that could be used to improve alignment.

While there was a near universal adoption of hierarchical sampling designs, almost no studies adjusted for clustering of infection at collection when conducting their analysis. The implications of this analytical omission are not trivial. Many studies aimed to compare their estimates (and confidence intervals) of prevalence to threshold values, either thresholds required for the WHO certification of elimination (onchocerciasis) or provisional thresholds (LF). Failure to account for clustering in the analysis of data from hierarchical surveys may have led to artificially narrow confidence intervals [100] and therefore, undue confidence that prevalence was below a specified threshold. Similarly, other comparisons of prevalence, for example before and after interventions, could equally be in question due to a failure to account for clustering of infection. The absence of software with the capability to easily adjust pool‐tested data for hierarchical survey designs likely explains this major analytical omission. The only publicly available software with this capability, PoolTestR [100], was published by some of the authors of this review, in 2021 towards the end of the review period. Prior to this, the vast majority of studies reported using the PoolScreen software [98]. In the small number of studies where there was only a single collection site [23] or only one collection site per study area [41], adjusting for clustering of infection at collection sites would not have been necessary; however, these sampling designs make it difficult to generalise findings at the sample sites to the broader study population. We recommend that, where feasible, MX studies should collect vectors across multiple collection sites. Population‐level estimates of prevalence should account for clustering of infection at these collection sites, using a random effect model, geostatistical model, or similar. When collection sites are selected in a multi‐stage process (e.g., first select villages, then select collections sites from selected villages), then analysis should account for clustering of infection at each stage of selection (e.g., at village and collection site). As an additional benefit, random effect and geostatistical models act to regularise estimates of prevalence for areas selected for collection (e.g., village) and at each collection site, improving precision and in many cases pulling estimates away from the extremes (0% and 100%). As of writing this review, the only open‐source software for random‐effect and geostatistical modelling with pool‐tested MX data is PoolTestR [100] and PoolTools [102], written by some of the authors of this review.

Only three studies reported considering design effects when choosing their sample sizes [21, 28, 72]. Compared to simple random sampling, hierarchical surveys provide less information for the estimation of population prevalence and less power to detect rare pathogens. It is therefore best practice to increase target sample sizes by a factor, called a ‘design effect’, to account for this loss of information [97]. In general, design effects are largest when infection status is highly correlated within collection sites or survey areas (i.e., high intra‐cluster correlation [ICC]) and when the number of units sampled from each collection site is large^1^. Pool‐testing also reduces information with the design effect depending on the pathogen prevalence, pool size and test sensitivity [103]. WHO guidelines for onchocerciasis elimination certification set target sample sizes but do not discuss the effects or suggest the use of design effects [32]. The two studies in this review that increased sample sizes using design effects [21, 72] did not justify their choice of design effect on statistical grounds, so it is difficult to judge whether the additional sampling effort was sufficient. However, given the substantial clustering of human infection within communities and households reported for LF and onchocerciasis, proper consideration of design effects may substantially increase sample size requirements. We recommend that future MX studies state clearly why key elements of the sample design have been chosen, including the pool size, the number of collection sites, the method of selecting collection sites and the sampling effort at each site measured either with target number pools/insects per collection site or the duration of collection activities and expected yield per collection site. Sample size calculations should be performed in the earliest design stage of any MX survey, and these should include design effects for pooled testing and hierarchical survey designs to ensure that sample sizes are sufficient to achieve the study goals. There are two barriers to widespread adoption of this approach. First, there are limited tools available to undertake these sample size calculations. Authors of this study have begun to fill this gap with a new open‐source R package, PoolPoweR, which provides sample size calculators suited to MX surveys and other hierarchical surveys with pool‐testing [104]. Second, any calculation of design effects needs an estimate of the ICC, ideally from a previous survey in the same population or a similar population with the same pathogen and vector species. However, ICCs have only rarely been reported for MX surveys and may vary by disease, vector species, country and progress towards elimination. ICCs can be estimated for each stage in a hierarchical survey design with the same tools used to adjust for hierarchical sampling designs in prevalence estimates [100, 102]. We recommend that future MX studies report estimates of ICCs at each stage of their hierarchical sampling designs to provide a basis for the appropriate design of future studies.

There was also generally insufficient justification of sampling strategies and pool sizes used in studies. Common design choices, such as the number of units in pools, appear to have been chosen primarily because others have made the same choice before, without any evidence to indicate that it was optimal for their study objectives based on statistical, laboratory, or practical grounds. Many studies formed pools by combining insects collected from multiple collection sites. Though there are techniques using results from mixed pools to attempt to estimate prevalence for each population from which individual samples were collected [105] the available open‐source software that can conduct these analyses [99, 106] cannot account for clustering of infection at collection sites. Moreover, forming pools of insects from multiple collection sites inevitably leads to the loss of information even if analysed correctly. We recommend that future MX studies aiming to estimate pathogen prevalence should avoid combining insects collected from different collection sites into pools; each pool should contain insects from a single collection site.

Many studies fixed the pool size to be used across the survey, for example, all pools contained exactly 20 insects. Studies that used a fixed pool size rarely stated what was done with remaining insects and suggest possible inefficiencies in the use of resources. If any remaining insects are not tested, this reduces the total sample size and discards potential information that could be gained by testing all insects. If sampling continues until a target number of insects are captured (e.g., nightly trapping until a quota is reached), then more sampling effort (e.g., trapping nights) will be dedicated towards collecting insects in the collection sites with lowest insect yields. In either case, this may be inefficient in settings where the largest cost component in the study is sample collection. Modern molecular diagnostics and software tools can easily handle a range of pool sizes and the authors of this review are not aware of any strong rationale for employing a fixed pool size. Therefore, rather than fixing the number and size of pools for each collection site, we recommend that surveys fix the collection effort (e.g., number of trapping days) to be expended at each collection site and test all collected vectors, while ensuring that pool sizes stay below the diagnostic‐specific maxima. When designing a survey under this approach, the number of collection sites required to achieve the target sample size can be estimated by considering the average yield of vectors expected at each collection site based on previous surveys or a small pilot survey.

Though a detailed assessment of whether the pool sizes were appropriate in each of the reviewed studies is beyond the scope of this review, in some studies, the pools may have been too large as nearly all the pools were positive [21]. Similarly, though we cannot comment more generally on the optimal number of pools per site and this information was not provided in most studies, some studies included very few (often only one) pools per collection site [32, 83]. With only a single observation (pool) per site, it would not be possible to estimate the degree of site‐level clustering of outcomes, which in turn would make it difficult to assess how estimates from samples could be generalised to unsampled sites. We recommend that future MX surveys carefully consider the statistical implications (loss of information) associated with large pools by consulting existing statistical guidance [33] or using a sample size calculator that considers the effect of pool size (e.g., PoolPoweR [104]). In particular, we recommend that for any survey where it will be necessary to estimate the degree of clustering (e.g., if trying to calculate confidence intervals for prevalence or compare prevalence to a threshold) at least two pools of insects should be tested from most or all sites.

In about one third of studies, none of the tested insects were positive for pathogen DNA. If one assumes (near) perfect sensitivity of the test, a negative pool implies that all the constituent insects would have also been negative if tested individually and therefore, statistical analysis does not require adjustment for testing in pools. However, in surveys attempting to validate elimination status of a disease by comparing prevalence estimates and their confidence intervals to a threshold, there is still a need to adjust for hierarchical sampling designs to ensure that widths of confidence intervals are not underestimated. None of the studies with all negative samples adjusted for hierarchical sampling design in their analysis, and the only software used to adjust for hierarchical sampling designs in MX studies [100] remains to be validated in such a setting. However, there is a fundamental difficulty in estimating the degree of clustering of infection from a dataset with no evidence of infection. If the degree of clustering of infection is estimated in a wide range of MX studies from around the world as we have recommended above, these could be used as a prior to inform estimates of clustering (Bayesian paradigm) or as assumed values for computing confidence intervals (frequentist paradigm) when analysing data with no positives, or evaluating survey designs before sampling commences.

In many studies, data collected from collection sites (e.g., households) across smaller geographical units (e.g., sentinel villages) were combined to estimate prevalence for a larger geographical area (e.g., region/state/focus). Some studies used population representative methods such as random selection of villages with probability proportional to human population size [7, 17, 21, 78]. However, as many studies either did not state how the smaller geographical units were selected or selected them purposively [47, 52, 55, 62, 82, 90, 93], it is unclear whether data from the smaller geographical units could be validly combined to get unbiased estimates of prevalence in the larger geographical areas. This is not a problem for studies where the primary aim was to compare collection and laboratory methods [68, 82]; however, it may introduce biases when comparing prevalence between two samples, for example, before and after interventions [12, 16, 22, 42, 52, 53, 62, 90]. Many studies used MX to evaluate elimination status after many rounds of MDA [12, 42, 52, 62], in which case there was an obvious case to be made for selecting the highest risk sites for sampling. However, this approach is at odds with a threshold‐based approach as currently required by the WHO for onchocerciasis, where an estimate of population prevalence (implying a population‐representative survey) is required to compare to the target threshold. We recommend that future MX studies clearly state how collection sites are selected, including details of randomisation. When collection sites are selected purposively, we recommend that studies describe the reasons for selection (e.g., ease of access, previously high prevalence, abundant vector population) so that the implications for sampling bias are clear. We also recommend any study intending to purposively select collection sites should also select some sites using population representative methods (e.g., random, systematic, or spatially regulated sampling). This approach could reveal any systematic differences between purposive and population representative samples and allow unbiased estimates of prevalence to be obtained with appropriate methods [107].

Though there are published WHO guidelines for the use of MX for onchocerciasis programmes [32, 108] and provisional WHO guidelines for the role of MX and entomology for LF programmes [33, 34, 109], they do not and cannot include suggested designs that consider the resources and contextual constraints of each study or surveillance programme. Moreover, these guidelines and survey designs recommended in them have focused on post‐intervention settings where prevalence is low and the goal is to compare prevalence to a threshold. In particular, they do not advise on sample sizes and pool sizes required for other settings (e.g., high to moderate prevalence settings before and during interventions) or objectives (e.g., comparing prevalence between two samples). Tools that enable MX practitioners to evaluate a wide range of MX designs and identify those best suited to their study objectives and constraints could fill this gap. Though there is an extensive literature and many publicly available software tools for selecting and evaluating the statistical properties (e.g., power, sample size calculations) suited for hierarchical sampling designs, there is a major gap for hierarchical surveys using pooled testing. We have begun to fill this gap with a new R package, PoolPoweR [104]; however, there is no widely validated and accepted software, which provides all required tools.

There is an extensive literature and numerous software applications dedicated to the analysis of either pool‐tested data or hierarchical sampling data. However, little has been written about surveys that use pooling and hierarchical sampling designs [110, 111, 112]; software that can analyse these surveys has only recently become available [100], and these tools still have gaps. For instance, many studies in this review compared prevalence between two or more samples, but did not quantify the differences or conduct formal tests for the significance of these differences with methods that accounted for the hierarchical sampling and pool‐testing designs. Subramanian et al. highlighted the lack of suitable statistical tools for such analyses [20]. A subsequent study [21] used a regression model with the PoolTestR software [100] (published 2021) to make these comparisons; however, tools that simplify these comparisons may widen the use of these types of analyses, important for evaluating interventions or confirming trends. LF and onchocerciasis are acknowledged to be focal diseases [113, 114]. However, none of the studies in the review used a spatial framework to design or analyse their data. Spatial sampling and analysis schemes can substantially reduce the sample size required to estimate population prevalence [115]. A geospatial modelling framework has been developed and applied for MX for tick‐borne disease surveillance [116], but these models assume that all positive pools of vectors are retested to determine the infection status of individual vectors and are therefore not applicable to the vast majority of MX survey designs considered in this review. Applicable Bayesian geostatistical modelling approaches are possible with the PoolTestR software [100]; however, there are no published studies that demonstrate this kind of analysis with field data.

The final design and implementation of any MX survey must be uniquely tailored to the disease, setting and survey objective and comply with constraints set by the available resources, and by the practical logistics of field work. Nevertheless, there has been an apparent tendency amongst researchers to neglect several important aspects of survey design unique to the hierarchal surveys and pooled data analysis that are commonly employed in MX surveillance for NTDs. The failure to consider and adjust for the implications of hierarchical sampling designs on estimated prevalence is likely perpetuated by the paucity of examples in the literature which do so, and a lack of freely available and easy‐to‐use tools that facilitate the analysis. Such examples and tools are urgently needed in the MX space to improve the quality of the information being provided to inform major programmatic decisions on disease elimination.

## Conflicts of Interest

The authors declare no conflicts of interest.

## Supporting information


**Data S1:** tmi70017‐sup‐0001‐Tables.docx.

File A.

## Endnotes

^1^
A common formula for the design effect (D) in cluster surveys (i.e., a hierarchical survey with only one stage before selecting units) with individually tested units (i.e., without pooled testing) is $D=1+\left(n-1\right)\times \mathrm{ICC}$, where n is the number of units per cluster/collection site (Kish, 1965). The design effect can be substantial even if *ICC* is small if *n* is also large. For example, if n=100 and ICC=0.01, then D=1.99, that is, the target sample size needs to be doubled to account for clustering. If insects are tested in pools, the design effect will be even larger.

## References

[1] Y. Lin , K. Fang , Y. Zheng , H. L. Wang , and J. Wu , “Global Burden and Trends of Neglected Tropical Diseases From 1990 to 2019,” Journal of Travel Medicine 29, no. 3 (2022): 1–11. 10.1093/jtm/taac031 35238925

[2] World Health Organization , Ending the Neglect to Attain the Sustainable Development Goals: A Road Map for Neglected Tropical Diseases 2021–2030 (World Health Organization, 2020).

[3] A. M. Cadavid Restrepo , Predictive Risk Mapping of Lymphatic Filariasis Residual Hotspots in American Samoa Using Demographic and Environmental Factors (Cold Spring Harbor Laboratory, 2022). 10.1371/journal.pntd.0010840 PMC10399813 37486947

[4] J. R. Harris and R. E. Wiegand , “Detecting Infection Hotspots: Modeling the Surveillance Challenge for Elimination of Lymphatic Filariasis,” PLoS Neglected Tropical Diseases 11, no. 5 (2017): e0005610. 28542274 10.1371/journal.pntd.0005610 PMC5453617

[5] M. N. Katabarwa , I. M. A. Zarroug , N. Negussu , et al., “The Galabat‐Metema Cross‐Border Onchocerciasis Focus: The First Coordinated Interruption of Onchocerciasis Transmission in Africa,” PLoS Neglected Tropical Diseases 14, no. 2 (2020): e0007830. 32027648 10.1371/journal.pntd.0007830 PMC7004312

[6] C. L. Lau , S. Sheridan , S. Ryan , et al., “Detecting and Confirming Residual Hotspots of Lymphatic Filariasis Transmission in American Samoa 8 Years After Stopping Mass Drug Administration,” PLoS Neglected Tropical Diseases 11, no. 9 (2017): e0005914. 28922418 10.1371/journal.pntd.0005914 PMC5619835

[7] R. U. Rao , S. D. Samarasekera , K. C. Nagodavithana , et al., “Comprehensive Assessment of a Hotspot With Persistent Bancroftian Filariasis in Coastal Sri Lanka,” American Journal of Tropical Medicine and Hygiene 99, no. 3 (2018): 735–742. 30014812 10.4269/ajtmh.18-0169 PMC6169179

[8] N. Pilotte , T. R. Unnasch , and S. A. Williams , “The Current Status of Molecular Xenomonitoring for Lymphatic Filariasis and Onchocerciasis,” Trends in Parasitology 33, no. 10 (2017): 788–798. 28756911 10.1016/j.pt.2017.06.008

[9] Y. I. Coulibaly , S. Y. Coulibaly , H. Dolo , et al., “Dynamics of Antigenemia and Transmission Intensity of Wuchereria Bancrofti Following Cessation of Mass Drug Administration in a Formerly Highly Endemic Region of Mali,” Parasites & Vectors 9, no. 1 (2016): 628. 27912789 10.1186/s13071-016-1911-9 PMC5135747

[10] D. K. de Souza , S. Sesay , M. G. Moore , et al., “No Evidence for Lymphatic Filariasis Transmission in Big Cities Affected by Conflict Related Rural‐Urban Migration in Sierra Leone and Liberia,” PLoS Neglected Tropical Diseases 8, no. 2 (2014): e2700. 24516686 10.1371/journal.pntd.0002700 PMC3916318

[11] M. A. Dorkenoo , D. K. de Souza , Y. Apetogbo , et al., “Molecular Xenomonitoring for Post‐Validation Surveillance of Lymphatic Filariasis in Togo: No Evidence for Active Transmission,” Parasites & Vectors 11, no. 1 (2018): 52. 29361964 10.1186/s13071-017-2611-9 PMC5781303

[12] C. Botto , M. G. Basañez , M. Escalona , et al., “Evidence of Suppression of Onchocerciasis Transmission in the Venezuelan Amazonian Focus,” Parasites & Vectors 9 (2016): 40. 26813296 10.1186/s13071-016-1313-z PMC4728794

[13] L. Diawara , M. O. Traoré , A. Badji , et al., “Feasibility of Onchocerciasis Elimination With Ivermectin Treatment in Endemic Foci in Africa: First Evidence From Studies in Mali and Senegal,” PLoS Neglected Tropical Diseases 3, no. 7 (2009): e497. 19621091 10.1371/journal.pntd.0000497 PMC2710500

[14] A. A. Gbakima , M. A. Appawu , S. Dadzie , et al., “Lymphatic Filariasis in Ghana: Establishing the Potential for an Urban Cycle of Transmission,” Tropical Medicine & International Health 10, no. 4 (2005): 387–392. 15807803 10.1111/j.1365-3156.2005.01389.x

[15] A. B. Leite , A. R. Lima , R. B. Leite , et al., “Assessment of Family and Neighbors of an Individual Infected With Wuchereria Bancrofti From a Non‐Endemic Area in the City of Maceió, *Brazil* ,” Brazilian Journal of Infectious Diseases 14, no. 2 (2010): 125–128. 10.1590/s1413-86702010000200002 20563436

[16] F. Richards, Jr. , N. Rizzo , C. E. Diaz Espinoza , et al., “One Hundred Years After Its Discovery in Guatemala by Rodolfo Robles, Onchocerca Volvulus Transmission Has Been Eliminated From the Central Endemic Zone,” American Journal of Tropical Medicine and Hygiene 93, no. 6 (2015): 1295–1304. 26503275 10.4269/ajtmh.15-0364 PMC4674249

[17] I. O. Owusu , D. K. de Souza , F. Anto , et al., “Evaluation of Human and Mosquito Based Diagnostic Tools for Defining Endpoints for Elimination of Anopheles Transmitted Lymphatic Filariasis in Ghana,” Transactions of the Royal Society of Tropical Medicine and Hygiene 109, no. 10 (2015): 628–635. 26385935 10.1093/trstmh/trv070

[18] S. Isiyaku , M. Igbe , S. Madaki , et al., “The Interruption of Transmission of Onchocerciasis in Kaduna, Kebbi and Zamfara States, Nigeria: Another Milestone Achievement,” International Health 14, no. Suppl 2 (2022): ii43–ii54. 36130252 10.1093/inthealth/ihac036 PMC9492257

[19] L. J. Reimer , E. K. Thomsen , D. J. Tisch , et al., “Insecticidal Bed Nets and Filariasis Transmission in Papua New Guinea,” New England Journal of Medicine 369, no. 8 (2013): 745–753. 23964936 10.1056/NEJMoa1207594 PMC3835352

[20] S. Subramanian , P. Jambulingam , B. K. Chu , et al., “Application of a Household‐Based Molecular Xenomonitoring Strategy to Evaluate the Lymphatic Filariasis Elimination Program in Tamil Nadu, India,” PLoS Neglected Tropical Diseases 11, no. 4 (2017): e0005519. 28406927 10.1371/journal.pntd.0005519 PMC5404881

[21] B. McPherson , H. J. Mayfield , A. McLure , et al., “Evaluating Molecular Xenomonitoring as a Tool for Lymphatic Filariasis Surveillance in Samoa, 2018–2019,” Tropical Medicine and Infectious Disease 7, no. 8 (2022): 203. 36006295 10.3390/tropicalmed7080203 PMC9414188

[22] H. A. Farid , Z. S. Morsy , H. Helmy , R. M. R. Ramzy , M. el Setouhy , and G. J. Weil , “A Critical Appraisal of Molecular Xenomonitoring as a Tool for Assessing Progress Toward Elimination of Lymphatic Filariasis,” American Journal of Tropical Medicine and Hygiene 77, no. 4 (2007): 593–600. 17978055 PMC2196407

[23] R. A. Abong , G. N. Amambo , A. A. Hamid , et al., “The Mbam Drainage System and Onchocerciasis Transmission Post Ivermectin Mass Drug Administration (MDA) Campaign, Cameroon,” PLoS Neglected Tropical Diseases 15, no. 1 (2021): e0008926. 33465080 10.1371/journal.pntd.0008926 PMC7815102

[24] A. Ramesh , M. Cameron , K. Spence , et al., “Development of an Urban Molecular Xenomonitoring System for Lymphatic Filariasis in the Recife Metropolitan Region, Brazil,” PLoS Neglected Tropical Diseases 12, no. 10 (2018): e0006816. 30325933 10.1371/journal.pntd.0006816 PMC6203399

[25] S. Subramanian , P. Jambulingam , K. Krishnamoorthy , et al., “Molecular Xenomonitoring as a Post‐MDA Surveillance Tool for Global Programme to Eliminate Lymphatic Filariasis: Field Validation in an Evaluation Unit in India,” PLoS Neglected Tropical Diseases 14, no. 1 (2020): e0007862. 31978060 10.1371/journal.pntd.0007862 PMC7001988

[26] L. K. Hapairai , C. Plichart , T. Naseri , et al., “Evaluation of Traps and Lures for Mosquito Vectors and Xenomonitoring of Wuchereria Bancrofti Infection in a High Prevalence Samoan Village,” Parasites & Vectors 8 (2015): 287. 26016830 10.1186/s13071-015-0886-2 PMC4449966

[27] S. R. Irish , W. M. B. Stevens , Y. A. Derua , T. Walker , and M. M. Cameron , “Comparison of Methods for Xenomonitoring in Vectors of Lymphatic Filariasis in Northeastern Tanzania,” American Journal of Tropical Medicine and Hygiene 93, no. 5 (2015): 983–989. 26350454 10.4269/ajtmh.15-0234 PMC4703286

[28] A. Premkumar , A. N. Shriram , K. Krishnamoorthy , et al., “Molecular Xenomonitoring of Diurnally Subperiodic Wuchereria Bancrofti Infection in Aedes (Downsiomyia) Niveus (Ludlow, 1903) After Nine Rounds of Mass Drug Administration in Nancowry Islands, Andaman and Nicobar Islands, India,” PLoS Neglected Tropical Diseases 14, no. 10 (2020): e0008763. 33095805 10.1371/journal.pntd.0008763 PMC7641468

[29] R. U. Rao , S. D. Samarasekera , K. C. Nagodavithana , et al., “Programmatic Use of Molecular Xenomonitoring at the Level of Evaluation Units to Assess Persistence of Lymphatic Filariasis in Sri Lanka,” PLoS Neglected Tropical Diseases 10, no. 5 (2016): e0004722. 27196431 10.1371/journal.pntd.0004722 PMC4873130

[30] B. O'Neill and A. McLure , “An Examination of the Generalised Pooled Binomial Distribution and Its Information Properties,” 2021, arXiv Preprint arXiv:2108.04396.

[31] J. Katz and S. L. Zeger , “Estimation of Design Effects in Cluster Surveys,” Annals of Epidemiology 4, no. 4 (1994): 295–301. 7921319 10.1016/1047-2797(94)90085-x

[32] World Health Organization , Guidelines for Stopping Mass Drug Administration and Verifying Elimination of Human Onchocerciasis: Criteria and Procedures (World Health Organization, 2016). 26913317

[33] World Health Organization , “The Role of Polymerase Chain Reaction (PCR) Technique for Assessing LF Transmission,” Report of a Workshop, Copenhagen, Denmark, 2009, WHO/HTM/NTD/PCT.

[34] World Health Organization , Lymphatic Filariasis: A Handbook of Practical Entomology for National Lymphatic Filariasis Elimination Programmes (World Health Organization, 2013).

[35] World Health Organisation , Validation of Elimination of Lymphatic Filariasis as a Public Health Problem (World Health Organisation, 2017).

[36] J. Pryce and L. J. Reimer , “Evaluating the Diagnostic Test Accuracy of Molecular Xenomonitoring Methods for Characterising Community Burden of Lymphatic Filariasis,” Clinical Infectious Diseases 72, no. S3 (2021): S203–S209. 33906238 10.1093/cid/ciab197 PMC8201559

[37] J. Pryce , T. R. Unnasch , and L. J. Reimer , “Evaluating the Diagnostic Test Accuracy of Molecular Xenomonitoring Methods for Characterising the Community Burden of Onchocerciasis,” PLoS Neglected Tropical Diseases 15, no. 10 (2021): e0009812. 34637436 10.1371/journal.pntd.0009812 PMC8509893

[38] L. J. Reimer and J. D. Pryce , “The Impact of Mosquito Sampling Strategies on Molecular Xenomonitoring Prevalence for Filariasis: A Systematic Review,” Bulletin of the World Health Organization 102, no. 3 (2024): 204. 38420575 10.2471/BLT.23.290424 PMC10898278

[39] “Covidence Systematic Review Software,” Veritas Health Innovation, https://www.covidence.org.

[40] R Core Team , R: A Language and Environment for Statistical Computing (R Foundation for Statistical Computing, 2021).

[41] M. A. Adeleke , C. F. Mafiana , S. O. Sam‐Wobo , et al., “Biting Behaviour of Simulium Damnosum Complex and Onchocerca Volvulus Infection Along the Osun River, Southwest Nigeria,” Parasites & Vectors 3 (2010): 93. 20929573 10.1186/1756-3305-3-93 PMC2959032

[42] J. Convit , H. Schuler , R. Borges , et al., “Interruption of Onchocerca Volvulus Transmission in Northern Venezuela,” Parasites & Vectors 6, no. 1 (2013): 289. 24499653 10.1186/1756-3305-6-289 PMC3856516

[43] R. J. Gonzalez , N. Cruz‐Ortiz , N. Rizzo , et al., “Successful Interruption of Transmission of Onchocerca Volvulus in the Escuintla‐Guatemala Focus, Guatemala,” PLoS Neglected Tropical Diseases 3, no. 3 (2009): e404. 19333366 10.1371/journal.pntd.0000404 PMC2656640

[44] A. G. Guevara , J. C. Vieira , B. G. Lilley , et al., “Entomological Evaluation by Pool Screen Polymerase Chain Reaction of Onchocerca Volvulus Transmission in Ecuador Following Mass Mectizan Distribution,” American Journal of Tropical Medicine and Hygiene 68, no. 2 (2003): 222–227. 12641415

[45] Á. Guevara , R. Lovato , R. Proaño , et al., “Elimination of Onchocerciasis in Ecuador: Findings of Post‐Treatment Surveillance,” Parasites & Vectors 11, no. 1 (2018): 265. 29690907 10.1186/s13071-018-2851-3 PMC5937837

[46] A. Hendy , M. Krit , K. Pfarr , et al., “Onchocerca Volvulus Transmission in the Mbam Valley of Cameroon Following 16 Years of Annual Community‐Directed Treatment With Ivermectin, and the Description of a New Cytotype of Simulium Squamosum,” Parasites & Vectors 14, no. 1 (2021): 563. 34727965 10.1186/s13071-021-05072-y PMC8561987

[47] T. B. Higazi , I. M. A. Zarroug , H. A. Mohamed , et al., “Polymerase Chain Reaction Pool Screening Used to Compare Prevalence of Infective Black Flies in Two Onchocerciasis Foci in Northern Sudan,” American Journal of Tropical Medicine and Hygiene 84, no. 5 (2011): 753–756. 21540385 10.4269/ajtmh.2011.11-0009 PMC3083743

[48] M. N. Katabarwa , T. Lakwo , P. Habomugisha , et al., “Transmission of Onchocerca Volvulus Continues in Nyagak‐Bondo Focus of Northwestern Uganda After 18 Years of a Single Dose of Annual Treatment With Ivermectin,” American Journal of Tropical Medicine and Hygiene 89, no. 2 (2013): 293–300. 23690555 10.4269/ajtmh.13-0037 PMC3741251

[49] M. N. Katabarwa , P. Habomugisha , A. Khainza , et al., “Historical Elimination of Onchocerciasis From Victoria Nile Focus in Central Uganda Verified Using WHO Criteria,” American Journal of Tropical Medicine and Hygiene 102, no. 6 (2020): 1411–1416. 32228786 10.4269/ajtmh.20-0064 PMC7253126

[50] K. Komlan , P. S. Vossberg , R. G. Gantin , et al., “Onchocerca Volvulus Infection and Serological Prevalence, Ocular Onchocerciasis and Parasite Transmission in Northern and Central Togo After Decades of Simulium Damnosum s.l. Vector Control and Mass Drug Administration of Ivermectin,” PLoS Neglected Tropical Diseases 12, no. 3 (2018): e0006312. 29494606 10.1371/journal.pntd.0006312 PMC5849363

[51] K. A. Lindblade , B. Arana , G. Zea‐Flores , et al., “Elimination of Onchocercia Volvulus Transmission in the Santa Rosa Focus of Guatemala,” American Journal of Tropical Medicine and Hygiene 77, no. 2 (2007): 334–341. 17690408

[52] R. Lovato , A. Guevara , R. Guderian , et al., “Interruption of Infection Transmission in the Onchocerciasis Focus of Ecuador Leading to the Cessation of Ivermectin Distribution,” PLoS Neglected Tropical Diseases 8, no. 5 (2014): e2821. 24853587 10.1371/journal.pntd.0002821 PMC4031166

[53] R. S. Nicholls , S. Duque , L. A. Olaya , et al., “Elimination of Onchocerciasis From Colombia: First Proof of Concept of River Blindness Elimination in the World,” Parasites & Vectors 11, no. 1 (2018): 237. 29642939 10.1186/s13071-018-2821-9 PMC5896109

[54] L. C. Oforka , M. A. Adeleke , J. C. Anikwe , et al., “Biting Rates and Onchocerca Infectivity Status of Black Flies From the Simulium Damnosum Complex (Diptera: Simuliidae) in Osun State, *Nigeria* ,” Journal of Medical Entomology 57, no. 3 (2020): 901–907. 31901168 10.1093/jme/tjz250

[55] F. O. Richards , A. Eigege , J. Umaru , et al., “The Interruption of Transmission of Human Onchocerciasis by an Annual Mass Drug Administration Program in Plateau and Nasarawa States, *Nigeria* ,” American Journal of Tropical Medicine and Hygiene 102, no. 3 (2020): 582–592. 32043442 10.4269/ajtmh.19-0577 PMC7056427

[56] M. A. Rodríguez‐Pérez , R. Danis‐Lozano , M. H. Rodríguez , T. R. Unnasch , and J. E. Bradley , “Detection of Onchocerca Volvulus Infection in *Simulium ochraceum* Sensu Lato: Comparison of a PCR Assay and Fly Dissection in a Mexican Hypoendemic Community,” Parasitology 119, no. 6 (1999): 613–619. 10633923 10.1017/s0031182099005107

[57] M. A. Rodríguez‐Pérez , B. G. Lilley , A. Domínguez‐Vázquez , et al., “Polymerase Chain Reaction Monitoring of Transmission of Onchocerca Volvulus in Two Endemic States in Mexico,” American Journal of Tropical Medicine and Hygiene 70, no. 1 (2004): 38–45. 14971696

[58] M. A. Rodríguez‐Pérez , C. R. Katholi , H. K. Hassan , and T. R. Unnasch , “Large‐Scale Entomologic Assesment of Onchocerca Volvulus Transmission by Poolscreen PCR in Mexico,” American Journal of Tropical Medicine and Hygiene 74, no. 6 (2006): 1026–1033. 16760515

[59] M. A. Rodríguez‐Pérez , C. Lizarazo‐Ortega , H. K. Hassan , et al., “Evidence for Suppression of Onchocerca Volvulus Transmission in the Oaxaca Focus in Mexico,” American Journal of Tropical Medicine and Hygiene 78, no. 1 (2008): 147–152. 18187798

[60] M. A. Rodríguez‐Pérez , A. Domínguez‐Vázquez , T. R. Unnasch , et al., “Interruption of Transmission of Onchocerca Volvulus in the Southern Chiapas Focus, México,” PLoS Neglected Tropical Diseases 7, no. 3 (2013): e2133. 23556018 10.1371/journal.pntd.0002133 PMC3610615

[61] M. A. Rodríguez‐Pérez , H. Gopal , M. A. Adeleke , E. J. de Luna‐Santillana , J. N. Gurrola‐Reyes , and X. Guo , “Detection of Onchocerca Volvulus in Latin American Black Flies for Pool Screening PCR Using High‐Throughput Automated DNA Isolation for Transmission Surveillance,” Parasitology Research 112, no. 11 (2013): 3925–3931. 24030195 10.1007/s00436-013-3583-0

[62] M. A. Rodríguez‐Pérez , N. A. Fernández‐Santos , M. E. Orozco‐Algarra , et al., “Elimination of Onchocerciasis From Mexico,” PLoS Neglected Tropical Diseases 9, no. 7 (2015): e0003922. 26161558 10.1371/journal.pntd.0003922 PMC4498594

[63] M. O. Traore , M. D. Sarr , A. Badji , et al., “Proof‐of‐Principle of Onchocerciasis Elimination With Ivermectin Treatment in Endemic Foci in Africa: Final Results of a Study in Mali and Senegal,” PLoS Neglected Tropical Diseases 6, no. 9 (2012): e1825. 23029586 10.1371/journal.pntd.0001825 PMC3441490

[64] L. Yamèogo , L. Toè , J. M. Hougard , et al., “Pool Screen Polymerase Chain Reaction for Estimating the Prevalence of Onchocerca Volvulus Infection in Simulium Damnosum Sensu Lato: Results of a Field Trial in an Area Subject to Successful Vector Control,” American Journal of Tropical Medicine and Hygiene 60, no. 1 (1999): 124–128. 9988335 10.4269/ajtmh.1999.60.124

[65] I. M. Zarroug , K. Hashim , W. A. ElMubark , et al., “The First Confirmed Elimination of an Onchocerciasis Focus in Africa: Abu Hamed, Sudan,” American Journal of Tropical Medicine and Hygiene 95, no. 5 (2016): 1037–1040. 27352878 10.4269/ajtmh.16-0274 PMC5094213

[66] E. W. Chambers , S. K. McClintock , M. F. Avery , et al., “Xenomonitoring of Wuchereria Bancrofti and Dirofilaria Immitis Infections in Mosquitoes From American Samoa: Trapping Considerations and a Comparison of Polymerase Chain Reaction Assays With Dissection,” American Journal of Tropical Medicine and Hygiene 80, no. 5 (2009): 774–781. 19407123

[67] S. H. Cho , D. W. Ma , B. R. Koo , et al., “Surveillance and Vector Control of Lymphatic Filariasis in the Republic of Korea,” Osong Public Health and Research Perspectives 3, no. 3 (2012): 145–150. 24159506 10.1016/j.phrp.2012.07.008 PMC3738707

[68] Y. A. Derua , S. F. Rumisha , B. M. Batengana , et al., “Lymphatic Filariasis Transmission on Mafia Islands, Tanzania: Evidence From Xenomonitoring in Mosquito Vectors,” PLoS Neglected Tropical Diseases 11, no. 10 (2017): e0005938. 28985217 10.1371/journal.pntd.0005938 PMC5646871

[69] A. K. Dyab , L. A. Galal , A.‐S. Mahmoud , and Y. Mokhtar , “Xenomonitoring of Different Filarial Nematodes Using Single and Multiplex PCR in Mosquitoes From Assiut Governorate, Egypt,” Korean Journal of Parasitology 53, no. 1 (2015): 77–83. 25748712 10.3347/kjp.2015.53.1.77 PMC4384786

[70] M. E. Entonu , A. Muhammad , I. S. Ndams , and G. Franciosa , “Evaluation of Actin‐1 Expression in Wild Caught Wuchereria Bancrofti‐Infected Mosquito Vectors,” Journal of Pathogens 2020 (2020): 1–9. 10.1155/2020/7912042 PMC7547363 33062336

[71] H. A. Farid , R. E. Hammad , M. M. Hassan , et al., “Detection of Wuchereria Bancrofti in Mosquitoes by the Polymerase Chain Reaction: A Potentially Useful Tool for Large‐Scale Control Programmes,” Transactions of the Royal Society of Tropical Medicine and Hygiene 95, no. 1 (2001): 29–32. 11280059 10.1016/s0035-9203(01)90322-0

[72] S. R. Irish , H. M. al‐Amin , H. N. Paulin , et al., “Molecular Xenomonitoring for Wuchereria Bancrofti in *Culex quinquefasciatus* in Two Districts in Bangladesh Supports Transmission Assessment Survey Findings,” PLoS Neglected Tropical Diseases 12, no. 7 (2018): e0006574. 30048460 10.1371/journal.pntd.0006574 PMC6062013

[73] C. Jones , B. Ngasala , Y. A. Derua , et al., “Lymphatic Filariasis Transmission in Rufiji District, Southeastern Tanzania: Infection Status of the Human Population and Mosquito Vectors After Twelve Rounds of Mass Drug Administration,” Parasites & Vectors 11, no. 1 (2018): 588. 30424781 10.1186/s13071-018-3156-2 PMC6234578

[74] V. Khatri , N. Amdare , N. Chauhan , et al., “Epidemiological Screening and Xenomonitoring for Human Lymphatic Filariasis Infection in Select Districts in the States of Maharashtra and Karnataka, India,” Parasitology Research 118, no. 3 (2019): 1045–1050. 30666407 10.1007/s00436-019-06205-0 PMC6401222

[75] R. L. Korte , G. Fontes , J. D. S. A. A. Camargo , et al., “Survey of Bancroftian Filariasis Infection in Humans and Culex Mosquitoes in the Western Brazilian Amazon Region: Implications for Transmission and Control,” Revista da Sociedade Brasileira de Medicina Tropical 46, no. 2 (2013): 214–220. 23740057 10.1590/0037-8682-1708-2013

[76] B. L. Kouassi , D. K. de Souza , A. Goepogui , et al., “Assessing the Presence of Wuchereria Bancrofti in Vector and Human Populations From Urban Communities in Conakry, Guinea,” Parasites & Vectors 8 (2015): 492. 26410739 10.1186/s13071-015-1077-x PMC4583765

[77] C. L. Lau , K. Y. Won , P. J. Lammie , and P. M. Graves , “Lymphatic Filariasis Elimination in American Samoa: Evaluation of Molecular Xenomonitoring as a Surveillance Tool in the Endgame,” PLoS Neglected Tropical Diseases 10, no. 11 (2016): e0005108. 27802280 10.1371/journal.pntd.0005108 PMC5089733

[78] E. Lupenza , D. B. Gasarasi , and O. M. Minzi , “Lymphatic Filariasis, Infection Status in Culex Quinquefasciatus and Anopheles Species After Six Rounds of Mass Drug Administration in Masasi District, Tanzania,” Infectious Diseases of Poverty 10, no. 1 (2021): 20. 33648600 10.1186/s40249-021-00808-5 PMC7919328

[79] M. A. Moustafa , M. M. I. Salamah , H. S. Thabet , R. A. Tawfik , M. M. Mehrez , and D. M. Hamdy , “Molecular Xenomonitoring (MX) and Transmission Assessment Survey (TAS) of Lymphatic Filariasis Elimination in Two Villages, Menoufyia Governorate, Egypt,” European Journal of Clinical Microbiology & Infectious Diseases 36, no. 7 (2017): 1143–1150. 28155014 10.1007/s10096-017-2901-3

[80] E. Nchoutpouen , A. Talipouo , B. Djiappi‐Tchamen , et al., “Culex Species Diversity, Susceptibility to Insecticides and Role as Potential Vector of Lymphatic Filariasis in the City of Yaoundé, Cameroon,” PLoS Neglected Tropical Diseases 13, no. 4 (2019): e0007229. 30943198 10.1371/journal.pntd.0007229 PMC6464241

[81] D. D. Pam , D. K. de Souza , S. D'Souza , et al., “Is Mass Drug Administration Against Lymphatic Filariasis Required in Urban Settings? The Experience in Kano, Nigeria,” PLoS Neglected Tropical Diseases 11, no. 10 (2017): e0006004. 29020042 10.1371/journal.pntd.0006004 PMC5665554

[82] S. Pi‐Bansa , J. H. N. Osei , W. D. Kartey‐Attipoe , et al., “Assessing the Presence of Wuchereria Bancrofti Infections in Vectors Using Xenomonitoring in Lymphatic Filariasis Endemic Districts in Ghana,” Tropical Medicine and Infectious Disease 4, no. 1 (2019): 49. 30884886 10.3390/tropicalmed4010049 PMC6473662

[83] C. Plichart , Y. Sechan , N. Davies , and A. M. Legrand , “PCR and Dissection as Tools to Monitor Filarial Infection of Aedes Polynesiensismosquitoes in French Polynesia,” Filaria Journal 5, no. 1 (2006): 2. 16504131 10.1186/1475-2883-5-2 PMC1403774

[84] R. M. Ramzy , M. el Setouhy , H. Helmy , et al., “Effect of Yearly Mass Drug Administration With Diethylcarbamazine and Albendazole on Bancroftian Filariasis in Egypt: A Comprehensive Assessment,” Lancet 367, no. 9515 (2006): 992–999. 16564361 10.1016/S0140-6736(06)68426-2

[85] R. U. Rao , K. C. Nagodavithana , S. D. Samarasekera , et al., “A Comprehensive Assessment of Lymphatic Filariasis in Sri Lanka Six Years After Cessation of Mass Drug Administration,” PLoS Neglected Tropical Diseases 8, no. 11 (2014): e3281. 25393404 10.1371/journal.pntd.0003281 PMC4230885

[86] R. U. Rao , S. D. Samarasekera , K. C. Nagodavithana , et al., “Reassessment of Areas With Persistent Lymphatic Filariasis Nine Years After Cessation of Mass Drug Administration in Sri Lanka,” PLoS Neglected Tropical Diseases 11, no. 10 (2017): e0006066. 29084213 10.1371/journal.pntd.0006066 PMC5679644

[87] M. A. Schmaedick , A. L. Koppel , N. Pilotte , et al., “Molecular Xenomonitoring Using Mosquitoes to Map Lymphatic Filariasis After Mass Drug Administration in American Samoa,” PLoS Neglected Tropical Diseases 8, no. 8 (2014): e3087. 25122037 10.1371/journal.pntd.0003087 PMC4133231

[88] H. Takagi , T. C. Yahathugoda , B. Tojo , et al., “Surveillance of Wuchereria Bancrofti Infection by Anti‐Filarial IgG4 in Urine Among Schoolchildren and Molecular Xenomonitoring in Sri Lanka: A Post Mass Drug Administration Study,” Tropical Medicine and Health 47 (2019): 39. 31223271 10.1186/s41182-019-0166-5 PMC6567434

[89] V. Vasuki , S. L. Hoti , S. Subramanian , et al., “Multi‐Centric Evaluation of a Stage‐Specific Reverse Transcriptase‐Polymerase Chain Reaction Assay as a Xenomonitoring Tool for the Detection of Infective (L3) Stage Wuchereria Bancrofti in Vectors,” Indian Journal of Medical Research 154, no. 1 (2021): 132–140. 34782539 10.4103/ijmr.IJMR_713_19 PMC8715687

[90] G. J. Weil , W. Kastens , M. Susapu , et al., “The Impact of Repeated Rounds of Mass Drug Administration With Diethylcarbamazine Plus Albendazole on Bancroftian Filariasis in Papua New Guinea,” PLoS Neglected Tropical Diseases 2, no. 12 (2008): e344. 19065257 10.1371/journal.pntd.0000344 PMC2586652

[91] N. D. Wijegunawardana , Y. I. Gunawardene , A. Manamperi , H. Senarathne , and W. Abeyewickreme , “Geographic Information System (GIS) Mapping of Lymphatic Filariasis Endemic Areas of Gampaha District, Sri Lanka Based on Epidemiological and Entomological Screening,” Southeast Asian Journal of Tropical Medicine and Public Health 43, no. 3 (2012): 557–566. 23077834

[92] A. D. Wijegunawardana , N. S. Gunawardane , C. Hapuarachchi , et al., “Evaluation of PCR‐ELISA as a Tool for Monitoring Transmission of Wuchereria Bancrofti in District of Gampaha, Sri Lanka,” Asian Pacific Journal of Tropical Biomedicine 3, no. 5 (2013): 381–387. 23646302 10.1016/S2221-1691(13)60081-7 PMC3642448

[93] F. N. Yokoly , J. B. Z. Zahouli , A. Méite , et al., “Low Transmission of Wuchereria Bancrofti in Cross‐Border Districts of Côte D'ivoire: A Great Step Towards Lymphatic Filariasis Elimination in West Africa,” PLoS One 15, no. 4 (2020): e0231541. 32282840 10.1371/journal.pone.0231541 PMC7153895

[94] E. Michael , M. N. Malecela‐Lazaro , C. Kabali , L. C. Snow , and J. W. Kazura , “Mathematical Models and Lymphatic Filariasis Control: Endpoints and Optimal Interventions,” Trends in Parasitology 22, no. 5 (2006): 226–233. 16564745 10.1016/j.pt.2006.03.005

[95] E. M. Pedersen , W. A. Stolk , S. J. Laney , and E. Michael , “The Role of Monitoring Mosquito Infection in the Global Programme to Eliminate Lymphatic Filariasis,” Trends in Parasitology 25, no. 7 (2009): 319–327. 19559649 10.1016/j.pt.2009.03.013

[96] D. A. Boakye , H. A. Baidoo , E. Glah , C. Brown , M. Appawu , and M. D. Wilson , “Monitoring Lymphatic Filariasis Interventions: Adult Mosquito Sampling, and Improved PCR – Based Pool Screening Method for Wuchereria Bancrofti Infection in Anophelesmosquitoes,” Filaria Journal 6, no. 1 (2007): 13. 18047647 10.1186/1475-2883-6-13 PMC2235849

[97] L. Kish , Survey Sampling (John Wiley & Sons, 1965).

[98] C. R. Katholi and T. R. Unnasch , “Important Experimental Parameters for Determining Infection Rates in Arthropod Vectors Using Pool Screening Approaches,” American Journal of Tropical Medicine and Hygiene 74, no. 5 (2006): 779–785. 16687680

[99] B. Zhang , “binGroup: Evaluation and Experimental Design for Binomial Group Testing,” 2018.

[100] A. Mclure , B. O'Neill , H. Mayfield , C. Lau , and B. McPherson , “PoolTestR: An R Package for Estimating Prevalence and Regression Modelling for Molecular Xenomonitoring and Other Applications With Pooled Samples,” Environmental Modelling & Software 145 (2021): 105158.

[101] R. L. Wallace , D. M. Bulach , A. V. Jennison , et al., “Molecular Characterization of Campylobacter spp. Recovered From Beef, Chicken, Lamb and Pork Products at Retail in Australia,” PLoS One 15, no. 7 (2020): e0236889. 32730330 10.1371/journal.pone.0236889 PMC7392323

[102] F. Jaya and A. McLure , “PoolTools: Tools for Designing and Analysing Molecular Xenomonitoring Surveys and Other Data Tested in Pools,” 2024.

[103] X. M. Tu , E. Litvak , and M. Pagano , “On the Informativeness and Accuracy of Pooled Testing in Estimating Prevalence of a Rare Disease: Application to HIV Screening,” Biometrika 82, no. 2 (1995): 287–297.

[104] A. McLure and F. Jaya , “PoolPoweR: Power and Sample Size Calculations and Design Optimisaiton Tools for Surveys Using Pool Testing,” 2024.

[105] C. N. Joyner , C. S. McMahan , J. M. Tebbs , and C. R. Bilder , “From Mixed Effects Modeling to Spike and Slab Variable Selection: A Bayesian Regression Model for Group Testing Data,” Biometrics 76, no. 3 (2020): 913–923. 31729015 10.1111/biom.13176 PMC7944974

[106] B. Hitt , “binGroup2: Identification and Estimation using Group Testing,” 2020.

[107] E. Giorgi , S. S. S. Sesay , D. J. Terlouw , and P. J. Diggle , “Combining Data From Multiple Spatially Referenced Prevalence Surveys Using Generalized Linear Geostatistical Models,” Journal of the Royal Statistical Society: Series A (Statistics in Society) 178, no. 2 (2014): 445–464.

[108] World Health Organization , Certification of Elimination of Human Onchocerciasis: Criteria and Procedures (World Health Organization, 2001).

[109] World Health Organization , Defining the Roles of Vector Control and Xenomonitoring in the Global Programme to Eliminate Lymphatic Filariasis: Report of the Informal Consultation WHO/HQ, Geneva, 29‐31 January 2002 (World Health Organization, 2002).

[110] T. Birkner , I. B. Aban , and C. R. Katholi , “Evaluation of a Frequentist Hierarchical Model to Estimate Prevalence When Sampling From a Large Geographic Area Using Pool Screening,” Communications in Statistics—Theory and Methods 42, no. 19 (2013): 3571–3595. 24347808 10.1080/03610926.2011.633732 PMC3862083

[111] R. G. Clark , B. Barnes , and M. Parsa , “Clustered and Unclustered Group Testing for Biosecurity,” Journal of Agricultural, Biological and Environmental Statistics 29, no. 2 (2024): 193–211.

[112] P. Chen , J. M. Tebbs , and C. R. Bilder , “Group Testing Regression Models With Fixed and Random Effects,” Biometrics 65, no. 4 (2009): 1270–1278. 19210734 10.1111/j.1541-0420.2008.01183.x PMC2794992

[113] H. G. Zouré , M. Noma , A. H. Tekle , et al., “The Geographic Distribution of Onchocerciasis in the 20 Participating Countries of the African Programme for Onchocerciasis Control: (2) pre‐Control Endemicity Levels and Estimated Number Infected,” Parasites & Vectors 7, no. 1 (2014): 326. 25053392 10.1186/1756-3305-7-326 PMC4222889

[114] E. Michael , B. K. Singh , B. K. Mayala , M. E. Smith , S. Hampton , and J. Nabrzyski , “Continental‐Scale, Data‐Driven Predictive Assessment of Eliminating the Vector‐Borne Disease, Lymphatic Filariasis, in Sub‐Saharan Africa by 2020,” BMC Medicine 15, no. 1 (2017): 176. 28950862 10.1186/s12916-017-0933-2 PMC5615442

[115] P. J. Diggle , B. Amoah , C. Fronterre , E. Giorgi , and O. Johnson , “Rethinking Neglected Tropical Disease Prevalence Survey Design and Analysis: A Geospatial Paradigm,” Transactions of the Royal Society of Tropical Medicine and Hygiene 115, no. 3 (2021): 208–210. 33587142 10.1093/trstmh/trab020 PMC7946792

[116] R. Huang , A. C. McLain , B. H. Herrin , M. Nolan , B. Cai , and S. Self , “Bayesian Group Testing Regression Models for Spatial Data,” Spatial and Spatio‐Temporal Epidemiology 50 (2024): 100677. 39181610 10.1016/j.sste.2024.100677 PMC11347770

