# Supplementary material for: Current Gaps in Survey Design and Analysis for Molecular Xenomonitoring of Vector‐Borne Neglected Tropical Diseases: A Systematic Review
Source: Trop Med Int Health. 2025 Aug 5;30(9):893–907. doi: 10.1111/tmi.70017 (PMC12401650; doi:10.1111/tmi.70017)
Supplement: Supplementary file 1 — Data S1: tmi70017‐sup‐0001‐Tables.docx. [file TMI-30-893-s002.docx]

# Supplementary Materials

**Supplementary Table S1:**  Counts (%) papers per category that address each of the five most common objectives, noting that papers can have multiple objectives*.*

| **Objective** | **Onchocerciasis** | **LF** | **Overall** |
| --- | --- | --- | --- |
| Post-MDA elimination determination | 27(87) | 21 (47) | 48 (63) |
| Compare MX and human indicators | 18 (58) | 27 (60) | 45 (59) |
| Evaluate an intervention | 11 (35) | 8 (18) | 19 (25) |
| Compare collection methods | 0 (0) | 13 (29) | 13 (17) |
| Compare MX lab techniques | 5 (16) | 13 (29) | 18 (24) |

**Abbreviations** LF: lymphatic filariasis; MX: molecular xenomonitoring

**Supplementary Table S2**: Counts (%) of key design choices and justifications for the MX surveys in the included studies.

| **Design choice** | **Onchocerciasis** | **LF** | **Overall** |
| --- | --- | --- | --- |
| Hierarchical sampling frame | 29 (94) | 43 (96) | 72 (95) |
| Justified sample sizes | 9 (29) | 7 (16) | 16 (21) |
| Justified pool size | 2 (6) | 3 (7) | 5 (7) |
| Considered design effects in sample size calculations | 0 (0) | 2 (4) | 2 (3) |
| Same number of units in (nearly) all pools | 13 (42) | 17 (38) | 30 (39) |

**Abbreviations** LF: lymphatic filariasis

***Supplementary Table S3:***  Counts (%) of key analysis choices and outcomes for the MX surveys in the included studies.

| **Analysis choice or outcome** | **Onchocerciasis** | **LF** | **Overall** |
| --- | --- | --- | --- |
| All pools negative | 15 (48) | 9 (20) | 24 (32) |
| Estimation accounted for pooled testing | 30 (97) | 39 (87) | 69 (91) |
| Estimation accounted for clustering | 0 (0) | 1 (2) | 1 (1) |

**Abbreviations** LF: lymphatic filariasis
